# Impact of Bariatric Surgery on the Quality of Life of Obese Individuals with Chronic Low Back Pain

**DOI:** 10.1590/0100-6991e-20243706-en

**Published:** 2024-11-13

**Authors:** BRUNO ZIADE GIL, SIDNEY MORENO GIL, FLAVIO RAMALHO ROMERO

**Affiliations:** 1 - UNIFIPA - Faculdade de Medicina de Catanduva - Departamento de Cirurgia - Catanduva - SP - Brasil; 2 - Faculdade de Medicina de Botutcatu - Unesp, Professor do programa de PG em Anestesiologia - UNESP - Botutcatu - SP - Brasil

**Keywords:** Obesity, Inflammation Mediators, Low Back Pain, Bariatric Surgery, Quality of Life, Obesidade Mórbida, Cirurgia Bariátrica, Dor Lombar, Qualidade de Vida, Mediadores da Inflamação

## Abstract

**Introduction:**

Obesity has been increasing at alarming rates in Brazil and worldwide. It is known that bariatric surgery is an effective and safe treatment for severely obese adults with related comorbidities or morbidly obese individuals. Among the various comorbidities, chronic low back pain is one of the major sources of discomfort and reduced quality of life in these patients. Studies that investigate the impact of bariatric surgery on the lives of obese individuals with chronic low back pain are scarce.

**Methods:**

We analyzed 38 patients who were obese grade 3 or severely obese grade 2 and suffered from chronic low back pain. These patients underwent bariatric surgery. Eight variables were assessed before the surgery and 6 months post-surgery, comprising 5 quantitative variables and 3 qualitative variables.

**Results:**

Paired t-tests were used for the statistical analysis of quantitative variables. The mean values of interleukin-6 before the surgery did not differ statistically from post-operative measurements. However, the values of C-reactive protein, weight, BMI, and Oswestry Disability Index 2.0 were all statistically different post-operatively. As for qualitative variables, the 3 variables analyzed using the Wilcoxon test showed statistically significant differences.

**Conclusion:**

Substantial reduction in weight following bariatric surgery may be associated with significant reductions in chronic low back pain in the early post-operative period. This effect could result from an overall improvement in well-being associated with weight loss but may also be associated with a reduction in inflammatory factors, as indicated by the decrease in C-reactive protein, although not confirmed by interleukin-6.

## RESUMO

**Introdução:**

A obesidade vem aumentando em índices alarmantes no Brasil e em todo o mundo. Sabe-se que a cirurgia bariátrica é um tratamento eficaz e seguro para adultos obesos grau 2 com doenças relacionadas ou obesos grau 3. Entre as várias comorbidades, a dor lombar crônica é uma das que causam maior desconforto e perda de qualidade de vida nesses pacientes. Estudos que relacionam a o impacto da cirurgia bariátrica na vida de obesos portadores de lombalgia crônica são escassos.

**Métodos:**

Foram analisados 38 pacientes obesos grau 3 ou obesos grau 2, portadores de lombalgia crônica, submetidos à cirurgia bariátrica. Oito variáveis foram analisadas no pré-operatório e após 6 meses de cirurgia, sendo 5 quantitativas e 3 qualitativas.

**Resultados:**

O teste t pareado foi usado para análise estatística das variáveis quantitativas, sendo que a média dos valores de interleucina-6 no pré-operatório não foi estatisticamente diferente das dosagens no pós-operatório, porém os valores de PCR, peso, IMC e Índice Oswestry 2.0 de Incapacidade foram todos estatisticamente diferentes. Quanto às variáveis qualitativas, as 3 analisadas pelo teste de Wilcoxon obtiveram diferenças estatisticamente significativas.

**Conclusão:**

A redução substancial de peso após a cirurgia bariátrica pode estar associada a reduções importantes na lombalgia crônica no pós-operatório precoce. Este efeito pode ser o resultado de uma melhoria global no bem estar associada à perda de peso, porém também ter como associação na redução de fatores inflamatórios, demonstrado pela queda do PCR, porém não confirmado pela IL-6.

## INTRODUCTION

Obesity is as an abnormal or excessive accumulation of fat that poses a risk to health and well-being and is one of the most harmful medical conditions afflicting modern society, not only in terms of morbidity and mortality, but also in terms of costs, both for individuals and governments. Obesity is classified, according to the Body Mass Index (BMI), as grade 1 (BMI greater than or equal to 30 Kg/m^2^), grade 2 (BMI greater than or equal to 35 Kg/m^2^) and grade 3 or morbid (BMI greater than or equal to 40 Kg/m^2^). Currently, Obesity is an increasingly prevalent public health problem in Brazil and worldwide. Projections indicate that by 2023, 24.3% of Brazil’s adult population is expected to be obese (BMI of 30 Kg/m^2^ or higher), with women experiencing a slightly higher rate, at 24.8%. This represents an increase of just over 5% since 2016^1^. Obesity is strongly associated with type-2 diabetes mellitus, coronary artery disease, and obstructive sleep apnea. In addition, a BMI greater than 30 has been associated with a 50 to 100 percent increased risk of death from all causes compared with a BMI of 20 to 25. Obviously, obesity is associated with increased mortality and decreased overall health^2^. 

Low back pain corresponds to spinal and paraspinal symptoms in the lumbosacral region. There are several potential sources of low back pain, including, but not limited to, intervertebral discs, facet joints, vertebrae, neural structures, muscles, ligaments, and fascia^3^. Low back pain is also a common health problem, being a common cause of work-related disability. Low back pain is often classified and treated based on symptom duration, potential cause, presence or absence of radicular symptoms, and corresponding anatomical or radiographic abnormalities. Acute low back pain is defined as lasting less than four weeks, subacute low back pain lasts from four to 12 weeks, and chronic low back pain lasts for more than 12 weeks^4^. In the general population, the prevalence of acute low back pain ranges from 30% to 40%, the prevalence of subacute low back pain ranges from 25% to 60%, and chronic low back pain, from 10% to 13%. Low back pain is more common in women than in men^5^. 

Obese individuals have a higher prevalence of musculoskeletal pain and physical dysfunction than people of normal weight^6^. Several studies show a positive causal relationship between obesity and low back pain. Therefore, obese patients frequently receive recommendations to lose weight after experiencing low back pain for two primary reasons:

1) to reduce the mechanical load on the lumbar spine; and
2) to moderate the lordotic curvature induced by obesity in the lumbar spine^3^.

**Figure 1:**
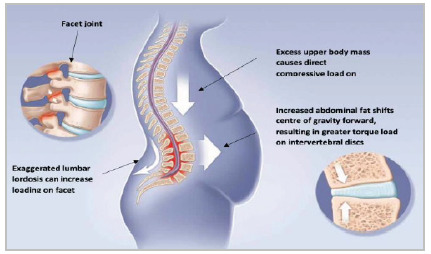
Obesity vs. low back pain: biomechanical causes (adapted).

**Figure 2:**
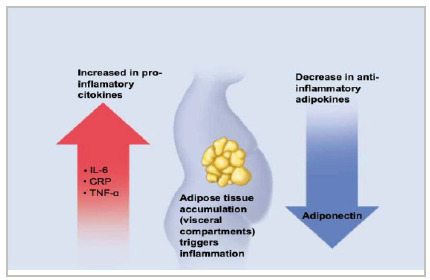
Obesity vs. low back pain: systemic inflammation (adapted).

A better understanding of the low back pain causative mechanisms may help with treatment options. However, the multifactorial nature of obesity and low back pain restricts the therapeutic resource of this disease, and studies exploring any causal effects between obesity and low back pain are lacking. Research has typically identified two main types of pathological mechanisms, 1) mechanical and 2) inflammatory^7^, illustrated in Figures 1 and 2 below. First, obesity could increase the mechanical load on the spine, causing a greater compressive force or greater shear on the lumbar spine structures during various activities. In addition, the increase in abdominal fat shifts the body’s center of gravity forward, resulting in greater load on the intervertebral discs. Consequently, obese individuals may compensate by exaggerating their lumbar lordosis, which in turn increases the load on the facet joints. Evidence of the second category of these pathological mechanisms is that some patients, even with a slight weight loss, report important improvements in low back pain. Therefore, there probably are non-biomechanical pathways involved. Due to the increase in systemic inflammation observed in the obese, some studies have found an association between inflammation and pain signaling and between low back pain and inflammation. In this context, many pro- and anti-inflammatory mediators that originate from or interact with adipose tissue and negatively affect both body fat and skeletal muscle have been studied. The main pro-inflammatory cytokines involved in this process are IL-6 and TNF-α, as well as C-Reactive Protein, a marker of systemic inflammation. Adiponectin, on the other hand, is the only adipokine that exerts anti-inflammatory effects on adipose tissue, macrophages, and skeletal muscle, and low levels of this adipokine are associated with high morbidity and high levels of C-Reactive Protein and IL-6^3^. This shows that changes in pain modulation through systemic inflammation can mediate low back pain. 

This study evaluates the association between changes in BMI induced by Bariatric Surgery and improvement in low back pain clinical symptoms. Since patients undergoing bariatric surgery often experience significant weight loss after surgery, they are an ideal population to evaluate the effects of weight reduction on low back pain. Thus, we followed a cohort of morbidly obese patients with chronic low back pain and undergoing bariatric surgery over a period of six months to evaluate the effect of weight loss on chronic low back pain and quality of life.

## METHODS

This is a longitudinal, prospective study, with data collected between June 2019 and October 2022. 

Obese patients with indication for Bariatric Surgery (BMI greater than or equal to 40 Kg/m^2^ and/or BMI greater than or equal to 35 Kg/m^2^ with comorbidities such as diabetes, hypertension, dyslipidemia, fatty liver disease associated with metabolic dysfunction, etc.) and with chronic low back pain were candidates for the study. Initially, 66 patients were selected according to the following inclusion criteria for the study: over 18 years of age, grade 3 obesity or grade 2 with comorbidities, with surgical indication and with a diagnosis of chronic low back pain, performed through anamnesis and physical examination. 

We excluded patients with severe cardiac or pulmonary comorbidities, neoplasms, individuals with diffuse pain syndromes (such as fibromyalgia), rheumatologic diseases (rheumatoid arthritis and ankylosing spondylitis), previous spine surgery, spinal cord trauma, and spinal infections (spondylodiscitis).

**Figure 3:**
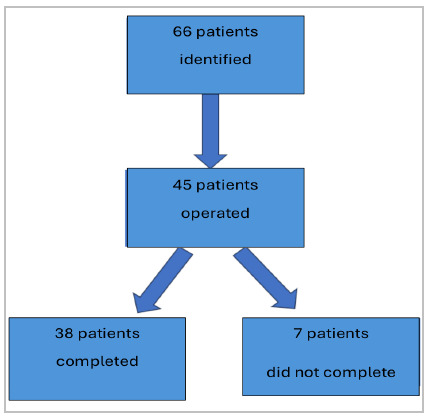
Flowchart of the conducted research.

According to the flowchart (Figure 3) below, we identified 66 patients who fit the inclusion criteria of the study. Of those, 45 were followed up for surgery. Of the 45 patients who underwent surgery, seven did not complete the six-month follow-up. All individuals signed an Informed Consent Form (ICF), thus agreeing to participate in the study.

The following data were obtained in the preoperative period: age, gender, current weight, height, BMI, presence or absence of associated diseases, bioimpedance data (lean mass, muscle mass, fat mass), inflammatory markers (CRP and IL-6), and impact of low back pain on quality of life through the Oswestry disability scale (ODI) and the SF-36 (Functional Capacity Scale Domains, Limitation of Physical Aspects and Pain). 

After surgery, the individuals were evaluated at 180 days for weight loss, BMI changes, inflammatory markers (CRP and IL-6), and repeated Oswestry and SF-36 scales (Functional Capacity Scale Domains, Limitation of Physical Aspects and Pain). 

The surgical techniques used were Laparoscopic Roux-en-Y Gastric Bypass, a mixed technique performed in 32 patients, and Laparoscopic Sleeve Gastrectomy, a restrictive surgery, performed in six patients.

This study was approved by the Ethics in Research Committee (CAAE: 21857219.2.3001.5411).

## RESULTS

We analyzed eight variables, five quantitative (Interleukin-6, CRP, weight, BMI, and Oswestry Disability Index 2.0) and three qualitative/categorical (SF 36 - Functional Capacity, SF 36 - Limitation by Physical Aspects, and SF 36 - Pain). 

Table 1 shows the demographic data, which included information on age (in years), sex (M/F), weight (in kg) and body mass index (BMI, in kg/m2). Table 2 covers categorical data in the pre and postoperative period, such as the results for the Functional Capacity Scale (SF-36), Limitation of Physical Aspects (SF-36), and Pain (SF-36), presented as minimum and maximum values, and the Oswestry disability index (%), as mean and standard deviation. Table 3 brings the laboratory data of C-Reactive Protein (CRP) and Interleukin-6 (IL-6) in the pre and postoperative periods, as mean and standard deviation. 

**Table 1:** Demographic and anthropometric data of patients in the pre and postoperative periods.

Data	Mean (SD)
Number of patients	38
Age (years)	32.39 (7.61)
Male (%)	3 (7.9)
Female (%)	35 (92.1%)
Preop weight (kg)	112.97 (17.47)
Postop weight (kg)	79.65 (13.06)
Preop BMI (kg/m2)	43.23 (5.35)
Postop BMI (kg/m2)	30.61(4.32)

**Table 2:** Data from the questionnaires answered in the pre and postoperative periods.

Data	Minimum value	Maximum Value
Preop SF 36 - Func Cap	5	85
Postop SF 36 - Func Cap	60	100
Preop SF 36 - Lim Phys Asp	0	100
Postop SF 36 - Lim Phys Asp	25	100
Preop SF 36 - Pain	0	62
Postop SF 36 - Pain	0	100
Preop Oswestry (%)	41.42 (17.10)	
Postop Oswestry (%)	2.53 (5.65)	

Func Cap: Functional Capacity; Lim Phys Asp: Limitation of Physical Aspects.

**Table 3:** Pre and postoperative laboratory data.

Data	Mean (SD)
Preop IL-6	3.71 (3.73)
Postop IL-6	3.72 (4.24)
Preop PCR (mg/L)	11.12 (8.38)
Postop PCR (mg/L)	5.74 (4.71)

Statistical analysis was conducted to assess the characteristics of the patients before and after the surgical intervention, as well as the differences in the measurements indicated by the analog scale, SF-36, and the Oswestry questionnaire. 

We used the paired t-test for differences between the means of quantitative variables, including IL-6 (pg/mL), CRP (mg/L), weight (kg), BMI (kg/m^2^) and Oswestry (%). For the metrics reported by the SF-36, such as Functional Capacity Scale, Limitation of Physical Aspects and Pain, we applied the Wilcoxon test to analyze the differences in medians before and after the surgical intervention. The level of significance adopted for all tests was 0.05.

**Graph 1:**
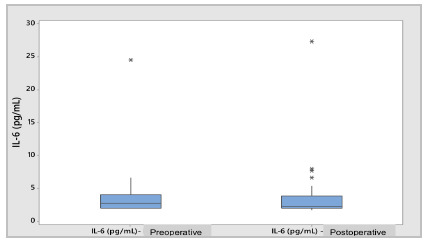
Box Plot - Preoperative vs. postoperative IL-6 (pg/mL).

The preoperative IL-6 variable, when compared to the postoperative period, did not present significant differences in the results (3.71±3.73 versus 3.72±4.24pg/mL; p=0.959), as shown in Graph 1 for the Box Plot of Differences.

**Graph 2:**
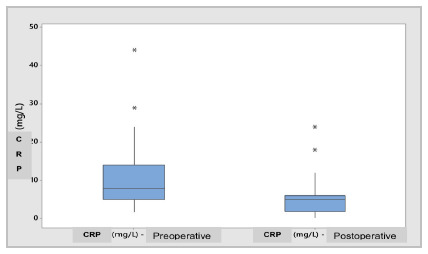
Box Plot - Preoperative vs. postoperative CRP (mg/L).

Also in relation to the laboratory measurements used in the study, the analysis of the CRP variable in the preoperative period when compared to the postoperative period showed significant differences in the results (11.12±8.38 versus 5.74±4.71mg/L; p=0.000), as shown in Graph 2 for the Box Plot of Differences.

Weight and BMI, in the preoperative period, when compared to the postoperative period, showed significant differences in the results (112.97±17.47 versus 79.65±16.06kg; p=0.000 and 43.23±5.35 versus 30.61±4.321kg/m^2^; p=0.000, respectively). 

**Graph 3:**
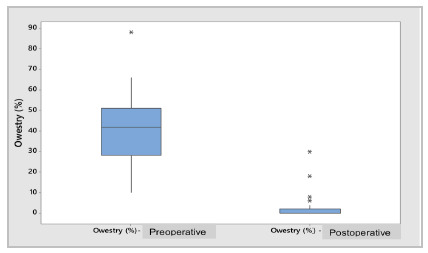
Box Plot - Preoperative vs. postoperative Oswestry (%)

**Graph 4:**
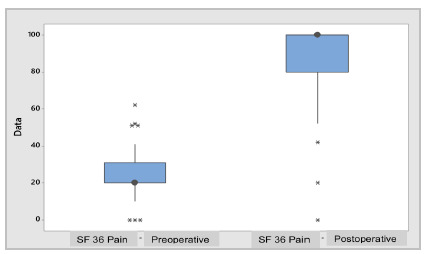
SBox Plot - Preoperative vs. postoperative SF 36 Pain.

Regarding the results of the questionnaires applied to patients in the pre and postoperative periods, the Oswestry (41.42±17.10 versus 2.53±5.65%; p=0.000 - Graph 3), SF 36 - Functional Capacity, SF 36 - Limitation by Physical Aspects and SF 36 - Pain (Graph 4) all presented statistically significant differences (p=0.000).

## DISCUSSION

**Figure 4:**
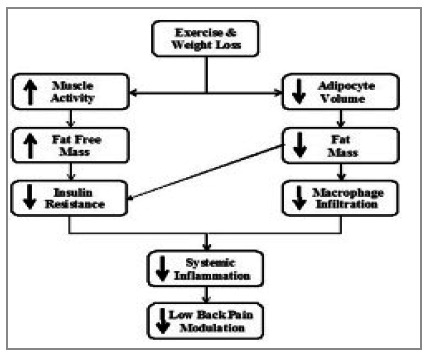
Relationship between Obesity and Low Back Pain (Adapted)

Several studies^3^ have demonstrated the relationship between obesity and chronic low back pain, and this relationship may go beyond simple weight loss, as shown in Figure 4. In addition, bariatric surgery may be the last treatment alternative for those patients with musculoskeletal limitations that prevent them from taking part in daily exercise programs to lose weight^9^ ^,^ ^10^. 

Two main pathological mechanisms have been listed. First, the mechanical overload on the lumbar spine, in addition to the compensatory hyperlordosis of the obese due to the previous change in the center of gravity; the second includes the increase in inflammatory cytokines, which creates a pro-inflammatory environment in obese patients, leading to a state of hyperalgesia^7^. An important systematic review of the literature^11^ demonstrated a substantial improvement in chronic low back pain in obese patients undergoing bariatric surgery, in addition to a significant improvement in the dysfunction of these patients, which was measured, among others, by two questionnaires (SF-36 Quality of Life and Oswestry Disability Index 2.0) used in our study. These results are similar to those we found. The hypotheses raised by the authors suggest that these improvements may be of a mechanical nature, directly related to the decrease in the load on muscles and joints and/or due to the subjective improvement of well-being and self-perception after surgery. 

A meta-analysis including seven studies^12^ showed a statistically significant improvement (p<0.001) in the SF-36 Quality of Life questionnaires and the Oswestry Disability Index 2.0 associated with a statistically significant decrease in BMI (p<0.001) postoperatively.

We also observed such findings, attesting to significant impact of the postoperative BMI reduction on the improvement in pain and quality of life questionnaires.

Still regarding quality-of-life improvement in the postoperative period of bariatric surgery, a study^14^ reported a significant improvement in the Visual Analogue Scale Questionnaire for Pain after six months of Sleeve Gastrectomy in women, data coinciding with ours, which showed significant results in the pain and quality of life scales in the postoperative period.

In another prospective observational study^13^, the authors demonstrated a significant decrease in serum levels of IL-6 and CRP (p<0.0001) measured in the pre and postoperative periods of 6 months of bariatric surgery, but they stated that the decrease in these inflammatory markers did not demonstrate a correlation with the improvement of pain and quality of life in the postoperative period. Statistical analysis of our data revealed interesting results. While IL-6 did not show significant differences before and after surgery, CRP did. This reduction in CRP may indicate a decrease in inflammation associated with chronic low back pain, showing a possible benefit of bariatric surgery in this context. This conflicting data in relation to IL-6 between our study and that of Richette et al. can be explained by the high sensitivity and low specificity of IL-6, which may have its values altered in various clinical conditions. Thus, our data suggest that this specific cytokine may not be directly affected by the procedure.

However, it is crucial to interpret the results in the context of overall quality of life. The significant reduction in CRP, even without changes in Interleukin-6, may indicate that other anti-inflammatory mechanisms or metabolic changes associated with surgery are contributing to the improvement of chronic low back pain and, consequently, quality of life, even in the early postoperative period, when there is no major weight loss. This study also highlights the importance of including subjective measures, such as quality of life questionnaires, to complement objective assessments. The use of the SF-36 and the ODI allows a more holistic understanding of the impact of bariatric surgery on the functional and emotional aspects of patients’ lives, as well as objective data.

This study contributes to clinical practice by highlighting not only the relationship between bariatric surgery and obesity, but also its impact on chronic low back pain. However, it is crucial to recognize the limitations of our study, such as the sample size, the lack of a control group, and the impossibility of blinded randomization. An important feature of our study is suggesting areas for future research, such as the investigation of other inflammatory markers. In addition, ethical considerations about bariatric surgery and its role in improving patients’ quality of life should be an integral part of the discussion. 

A specific approach to chronic low back pain is of great relevance, given the frequently observed association between obesity and musculoskeletal conditions. Much is discussed about biomechanical conditions associated with chronic low back pain in obese patients (hyperlordosis of the lumbar spine, excessive compression on joint structures, and anteriorization of the center of gravity), but there is little data on inflammatory issues related to the duet lumbar pain and obesity. Careful analysis of changes in inflammatory markers, such as CRP, can provide important clues about the inflammatory mechanisms present in the pathophysiology and benefits of bariatric surgery.

In summary, the findings of this study offer valuable insight into the impact of bariatric surgery on chronic low back pain, highlighting not only changes in inflammatory parameters but also improvements in quality of life.

## CONCLUSIONS

Bariatric surgery has a significant impact on improving postoperative pain and quality of life in obese individuals with chronic low back pain.

Serum levels of C-reactive protein were significantly lower in the postoperative period of obese individuals with chronic low back pain undergoing bariatric surgery. The same was not observed for serum Interleukin-6 levels.
